# Association of Acyl-Ghrelin With Posttraumatic Stress Disorder in Adolescents Who Experienced Severe Trauma

**DOI:** 10.1001/jamanetworkopen.2020.13946

**Published:** 2020-08-20

**Authors:** Muhammad Omar Malik, Mohsin Shah, Muhammad Irfan ul Akbar Yousufzai, Najeeb Ullah, JoColl A. Burgess, Ki Ann Goosens

**Affiliations:** 1 Department of Physiology, Institute of Basic Medical Sciences, Khyber Medical University, Peshawar, Pakistan; 2 Department of Psychiatry, Friedman Brain Institute, Center for Affective Neuroscience, Icahn School of Medicine at Mount Sinai, New York, New York

## Abstract

This cross-sectional study examines acyl-ghrelin levels in Pakistani adolescents who experienced severe trauma and have posttraumatic stress disorder (PTSD) compared with adolescents who have not experienced severe trauma.

## Introduction

Chronic stress, the occurrence of trauma and its adverse consequences over a prolonged period, is associated with the development of posttraumatic stress disorder (PTSD), even when many years have elapsed since the initial stress exposure.^1^ Acyl-ghrelin levels increase in rodents and humans during chronic stress exposure.^2,3^ Elevated acyl-ghrelin remains long after the primary stressful event ceases (months in rodents, years in humans). Acyl-ghrelin, which is released mostly by the gut during times of energy depletion,^4^ is the only form of ghrelin that can bind to the ghrelin receptor. In rodents, stress-related increases in acyl-ghrelin underlie a long-term vulnerability to excessive fear.^3,5^ Here, we sought to determine whether elevated levels of acyl-ghrelin were associated with the development of PTSD or its severity in adolescents who experienced severe trauma.

## Methods

This study was approved by the institutional review board of Khyber Medical University. All participants and their respective caregivers gave written informed consent. This study followed the Strengthening the Reporting of Observational Studies in Epidemiology (STROBE) reporting guidelines.

We conducted a cross-sectional study on 49 adolescents who experienced severe trauma and 39 healthy, matched control participants. Adolescents in the trauma group experienced a terror attack and were injured, or lost a parent, relative, or close friend. Adolescents in the control group had no terror-associated losses or injuries. Blood and saliva samples were collected for analyses of acyl-ghrelin and cortisol, respectively, and all participants were administered the PTSD CheckList–Civilian Version (PCL-C).

Statistical analyses were performed with Prism statistical software version 6.0 (GraphPad Software) and JMP Pro statistical software version 14 (SAS Institute). Statistical significance was determined by the Kruskal-Wallis test, Mann-Whitney *U *test, one-way ANOVA, Pearson χ^2 ^test, or Dunn multiple comparisons, as appropriate. For all tests, testing was 2-sided and adjusted *P* < .05 was considered significant. Recruitment began December 2014, and data collection was completed in July 2015. Statistical analyses were performed February 2019 to May 2020. Further details of the experiment and analyses are included in the eAppendix in the Supplement.

## Results

The 88 study participants had a mean (SD) age of 14.31 (2.81) years; 85 (96%) were male. Within the trauma group, 14 adolescents (36%) did not meet the criteria for PTSD; no adolescents met the criteria for PTSD in the control group (Table). In the trauma group, adolescents with or without PTSD did not differ from controls or each other, except for body mass index. Adolescents in the trauma group without PTSD had levels of acyl-ghrelin (mean [SD], 61.36 [26.22] pg/mL [to convert acyl-ghrelin to picomoles per liter, multiply by 0.3]) (Figure, panel A, and Table) and cortisol (mean [SD], 43.34 [25.79] ng/mL [to convert cortisol to micromoles per liter, multiply by 2.7]) (Table) that were statistically indistinguishable from those of adolescents in the control group (75.13 [47.38] pg/mL acyl-ghrelin and 41.52 [29.01] ng/mL cortisol), whereas adolescents in the trauma group with PTSD had higher levels of both hormones (acyl-ghrelin: mean [SD], 166.1 [93.19] pg/mL and cortisol: mean [SD], 140.4 [158.5] ng/mL; median [interquartile range] of 65 [32.5-185]). The PCL-C scores of adolescents with trauma but without PTSD (mean [SD], 38.71 [5.73]) were higher than those of adolescents in the control group (mean [SD], 19.67 [2.20]), but lower than those of adolescents with trauma and PTSD (mean [SD], 55.03 [6.42]) (Figure, panel B). Acyl-ghrelin alone accounted for 76.3% of the variability in PCL-C score (Figure, panel C). A regression analysis of the PCL-C scores in the trauma group that included acyl-ghrelin, morning cortisol, body mass index, age, and their 2-way interactions (*F*_10,36_ = 9.52; *P* < .001) accounted for only an additional 2.24% of the variability in PCL-C scores; only acyl-ghrelin retained a significant association with PCL-C score (B, 26.77; 95% CI, 18.16-34.68; *P* < .001). There was a significantly elevated risk of PTSD when comparing adolescents with trauma and low levels of acyl-ghrelin with those with moderately elevated levels (Figure, panel C) (odds ratio, 7.94; 95% CI, 1.60-39.42; χ^2^ = 6.43; *P* = .01). All adolescents in the trauma group with high levels of acyl-ghrelin had PTSD.

**Table.** Characteristics of the Study Population Stratified by PTSD Diagnosis

Characteristic	Value, mean (SD)			Group difference *P* value
	Control (n = 39)^a^	Trauma		
		Without PTSD (n = 14)	With PTSD (n = 35)	
Age, y	14.33 (2.64)	14.07 (3.25)	14.37 (2.88)	.98^b^
Time since trauma, y	NA	4.85 (1.41)	4.34 (1.82)	.45^c^
Body mass index^d^	20.33 (3.29)	17.32 (3.08)	17.88 (3.39)	.002^e^
Socioeconomic status^f^	2.69 (0.52)	2.57 (0.51)	2.68 (0.53)	.74^g^
Acyl-ghrelin, pg/mL	75.13 (47.38)	61.36 (26.22)	166.1 (93.19)	<.001^b^
Median (interquartile range)	75 (40-105)	49.5 (43-91.25)	135 (105-200)	
Morning cortisol, ng/mL	41.52 (29.01)	43.34 (25.79)	140.4 (158.5)	.02^b^
Median (interquartile range)	35 (18.5-65)	40 (25-70)	65 (32.5-185)	
PTSD CheckList–Civilian Version score	19.67 (2.20)	38.71 (5.73)	55.03 (6.42)	<.001^b^

Abbreviations: NA, not applicable; PTSD, posttraumatic stress disorder.

SI conversion factors: To convert acyl-ghrelin to pmol/L, multiply by 0.3; to convert cortisol to μmol/L, multiply by 2.7.

^a^ No subjects in the nontraumatized group met the criteria for PTSD. Thus, this group is not stratified and is considered the control group.

^b^ Kruskal-Wallis test.

^c^ Mann-Whitney *U* test.

^d^ Body mass index is calculated as weight in kilograms divided by height in meters squared.

^e^ One-way ANOVA.

^f^ Socioeconomic status was coded as 1 = lower class, 2 = middle class, 3 = upper class.

^g^ Pearson χ^2 ^test.

**Figure.**
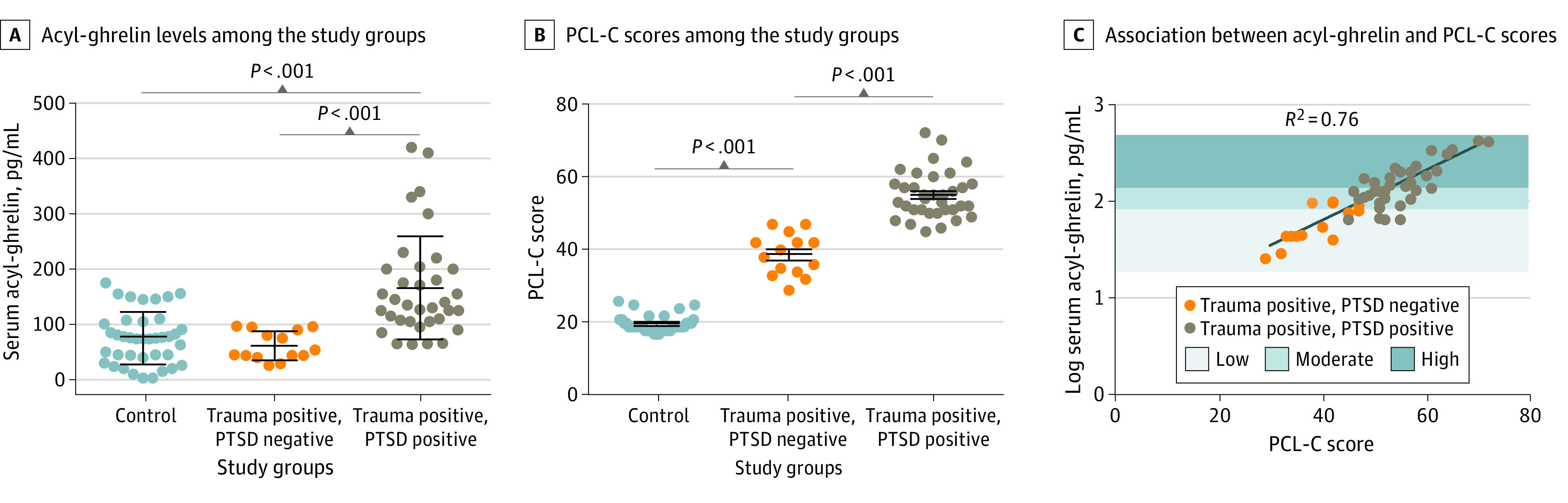
Association of Serum Acyl-Ghrelin With Posttraumatic Stress Disorder (PTSD) and Its Severity in Adolescents A and B, Each circle represents 1 participant. Vertical lines and error bars depict the median and interquartile range for each group. Corrected *P* values for Dunn multiple comparisons are shown. Dunn comparisons were used because the data were not normally distributed. C, The line represents the best-fit regression model (*P* < .001) of PTSD CheckList–Civilian Version (PCL-C) score by acyl-ghrelin concentration alone.

## Discussion

We observed an association between acyl-ghrelin and PTSD symptom severity in adolescents who experienced severe trauma. These data motivate additional research to investigate the mechanisms underlying trauma-associated elevation of acyl-ghrelin; these data also suggest that blood banks collecting samples from patients with PTSD should use methods that preserve acyl-ghrelin for analysis. This study’s limitations include the low number of female adolescents and the assessment of acyl-ghrelin at only a single posttrauma time point. It is unknown whether the adolescents who experienced severe trauma without PTSD never displayed trauma-associated elevation of acyl-ghrelin or whether acyl-ghrelin decreased over time since the initial trauma. It is also of interest to determine whether obesity, which dysregulates acyl-ghrelin,^6^ alters the role of acyl-ghrelin in stress and trauma.

## Notes

Supplement. **eAppendix.** Supplemental Methods **eReferences**
