# Supplementary material for: Association of Acyl-Ghrelin With Posttraumatic Stress Disorder in Adolescents Who Experienced Severe Trauma
Source: JAMA Netw Open. 2020 Aug 20;3(8):e2013946. doi: 10.1001/jamanetworkopen.2020.13946 (PMC7441359; doi:10.1001/jamanetworkopen.2020.13946)
Supplement: Supplement. — eAppendix. Supplemental Methods eReferences [file jamanetwopen-3-e2013946-s001.pdf]

Supplementary Online Content

Malik MO, Shah M, Irfan ul Akbar Yousufzai M, Ullah N, Burgess JA, Goosens KA. Association of acyl-ghrelin with posttraumatic stress disorder in adolescents who experienced severe trauma. *JAMA Netw Open*. 2020;3(8):e2013946. doi:10.1001/jamanetworkopen.2020.13946

eAppendix. Supplemental Methods

eReferences

This supplementary material has been provided by the authors to give readers additional information about their work.

© 2020 Malik MO et al. *JAMA Network Open.*

eAppendix

Supplemental Methods

We previously showed that this population of traumatized adolescents had elevated acyl-ghrelin relative to non-traumatized, matched controls [1]. Here, we extend these results by reporting cortisol measurements and stratifying the population according to PTSD diagnosis. The study was approved by the Institutional Review Board of Khyber Medical University, Pakistan (Study Approval No. ASRB000231/SC/IBMS).

Subject Recruitment

Participants (n = 101) were recruited from a densely settled area of Peshawar, located in the northwest part of Khyber Pukhtunkhwa province, a geographic locale affected by terrorist activities for more than a decade. The minimum size of the recruited groups (Traumatized and Control; n = 40 each) was determined *a priori*. Families with children who had lost a family member or were injured in terror attacks were identified through consultation with the local 'Jirga' (councils of elders). Researchers used local social contacts to identify families from the same neighborhoods as the participants identified by the Jirga. Researchers arranged for study visits with all parties that expressed an interest in the study. Samples were collected and questionnaires were administered at the homes of the participants.

Subject Exclusions

Children previously diagnosed with any mental illness or a developmental disorder were excluded from the study. Study participants taking antidepressants, antipsychotic drugs or anti-anxiety drugs were excluded from the study, as were children who had either previously suffered

from significant illness (Dengue Fever, etc.) or who currently showed signs of acute or chronic illness by physical examination. Thirteen children were excluded from the study for these reasons; no data was included from these subjects in any analysis.

Salivary Sample Collection

Some subjects (n = 37 traumatized, n = 23 control) consented to the collection of an early morning saliva sample to measure cortisol (at 8 A.M.). Some subjects may not have chosen to participate in this portion of the study because of the early time, or because it extended the time commitment required such that the study visit spanned a full day. These participants were asked not to exercise or eat for at least 30 min prior to saliva collection. At the time of sample collection, subjects were instructed to place a cotton ball in their mouth, roll it with their tongue, and gently chew for 1 min to saturate the cotton ball. The saturated cotton ball was squeezed into a sterile microfuge tube. The tube was rapidly transferred to a -80°C freezer until analysis. The analysis of each saliva sample was performed within one month of collection.

Demographic and Clinical Data Collection

For all subjects (n = 49 traumatized, n = 39 control), a structured, face-to-face interview was performed on the afternoon of the study visit. Subjects were instructed to not eat after lunch so that a fasting venous blood sample could be collected. Children and their caregivers were both present throughout the visit. Questionnaires were administered in Urdu and were translated to English. All subjects received venipuncture to collect a blood sample for the analysis of acyl-ghrelin (at 5 P.M). During a clinical evaluation, occurring immediately before or after venipuncture, all subjects were administered the PTSD CheckList—Civilian Version (PCL-17; hereafter referred to as PCL) translated into Urdu by four experimenters fluent in both Urdu and

English (see **Supplement, Questionnaire**). This questionnaire has a high correlation with the results of the Clinician-Administered PTSD Scale—Child/Adolescent Version (CAPS-CA) [2]. Anthropomorphic and health information for each child was collected during the interview. The questions addressed changes in health status since the time of the terror attack that resulted in the death of a loved one or injury to the child (for children in the Traumatized groups) and changes in the health status since the time of the natural death of a loved one (if applicable) or in recent years (for children in the Control group). Additionally, parents were asked to provide information about their education levels, occupations, and household income (in Pakistani rupees).

Venous Blood Sample Collection

The protease inhibitor 4-(2-aminoethyl) benzenesulfonyl fluoride hydrochloride (AEBSF; 200 mg/ml) was mixed in distilled water and maintained on ice prior to sample collection. A small volume of blood (3 ml) was drawn by trained research staff. Within two minutes of blood collection, aliquots of blood (100 µl) were generated; AEBSF was added to each aliquot at a 1:100 dilution (i.e. 1 µl AEBSF per 100 µl of blood). Samples were gently inverted five times to mix completely, and then placed on ice. Serum was then collected from the clotted blood aliquots and stored at -80°C until analysis.

Processing of Saliva and Serum Samples

For salivary samples, a salivary cortisol ELISA kit (CO116S; Calbiotech Inc., 10461 Austin Dr, Spring Valley, CA) was used. Acyl-ghrelin was measured using an ELISA kit as previously described [1]. For all ELISAs, the results were analyzed by experimenters not involved in the sample collection who were blinded to the group information associated with the sample.

Samples were run in duplicate and mean values were used in subsequent analyses. All reported values were dilution-corrected.

PCL Scoring

For the PCL, a total score was created by summing the responses across all 17 items. Responses to individual items were classified as symptomatic if rated at "3: Moderately" or above. Subjects were classified as having PTSD (PTSD+) if they were symptomatic for one or more Re-experiencing items (items 1-5), three or more Avoidance/Numbing items (items 6-12), and two or more Arousal items (items 13-17). All subjects that were not classified as PTSD+ were classified as not having PTSD (PTSD-).

Socioeconomic Status Scoring

To calculate socioeconomic status, the Kuppuswamy classification scale was used [3]. The Kuppuswamy classification scale is the most commonly used measure of socioeconomic status in Pakistan and is updated regularly to index the income scales to changes in the consumer price index for Pakistan. This scale assigns a numerical score to the educational level attained by the heads of the household, the occupations of the heads of the household, and the household income. These three scores were summed to generate a total score. The total scores were converted to class ratings (upper class: 26-29; middle class: 11-25; lower class: 3-10). These ratings were recoded as categorical variables (upper class = 3, middle class = 2, lower class = 1).

Statistics

Body Mass Index [BMI] was computed by dividing body mass (kg) by height squared (m^2).

Statistical outliers for each measure were identified as values that were more than three standard deviations from the group mean. One data point was excluded from analysis of the Morning Cortisol for this reason (from the Traumatized, PTSD+ Group); no other outliers were identified. Normality of data was analyzed with the D'Agostino & Pearson omnibus normality test. Group means of continuous variables from a normal distribution were compared using one-way ANOVA followed by Tukey's multiple comparisons. Group means of continuous variables that were not normally distributed were compared using either the Kruskal-Wallis test (for three-group comparisons), followed by Dunn's multiple comparisons corrected for the number of comparisons, or the two-tailed Mann Whitney test (for two-group comparisons). Group means of categorical variables were compared using Pearson Chi Square Tests.

Least squares linear regression was used to examine the association between PCL score and acyl-ghrelin (Figure, c). To determine whether variability in the PCL score could be better accounted for with additional variables, we used multivariable least squares linear regression to examine the associations between PCL scores and acyl-ghrelin, morning cortisol, age, body-mass index, and their full interactions in the traumatized population. An initial regression model suggested that all three- and four-way interactions were likely redundant with other factors in the model [indicated by Variance Inflation Factors (VIFs) ranging from 5 to 10]. To create a parsimonious model and minimize overfitting of the model, these redundant interactions were removed. The terms of the resulting model all had VIF values less than 2, indicating that the factors and their interactions were not correlated (low multicollinearity). For these linear regressions, acyl-ghrelin, morning cortisol, and age values were log-transformed (base 10) to meet normality requirements for the regression.

To quantify the elevated risk of PTSD associated with elevated acyl-ghrelin, we performed an odds ratio (OR) analysis. Each traumatized subject was classified as having low, moderate, or high levels of acyl-ghrelin. The distribution of acyl-ghrelin values in the traumatized population was divided into thirds, with an equal number of subjects in each third. The lowest third of values were classified as "low, the middle third as "moderate", and the upper third as "high". A logistic regression was run to determine the association of the low and moderate categorical acyl-ghrelin values with the binary outcome of PTSD diagnosis (Yes/No). An odds analysis for subjects with high acyl-ghrelin levels was not performed because all subjects with high ghrelin levels had PTSD (the odds ratio for this group is infinity).

Questionnaire

The PCL-17-C was translated into Urdu and is provided below. The English version of the PCL-17-C was developed by the Veterans Affairs National Center for PTSD and is also provided below.

PTSD Checlist- Civilian Version (PCL-C) Urdu Version

نیچے دیے گئے سوالوں حالات کو غور سے پڑھیں اور آپ بیان کی گئی حالت سے جس قدر پریشان ہیں اس کے سامنے والے خانے میںχ کا نشان لگائیں

انتہائی (5) زیادہ	تھوڑا (4) زیادہ	اعتدال (3) سے	تھوڑا (2) بہت	بالکل (1) نہیں	سوال/حالت	نمبر
					ماضی کی کسی تکلیف دہ سوچ، یادیں، یا پریشان کن تجربہ کا بار بار یاد آنا	1
					ماضی کے کسی پریشان کن خواب کا بار بار آنا	2
					اچانک کسی تکلیف دہ تجربہ کو دوبارہ محسوس کرنا یا سامنا کرنا جبکہ آپ اس سے مطمئن ہو چکے ہوں	3
					ماضی کے کسی تکلیف دہ یا تلخ تجربہ کا آپ کو بے حد پریشان کرنا	4
					کوئی جسمانی رد عمل جیسے دل کا زور سے دھڑکنا، سانس لینے میں تکلیف ہونا، یا پسینے آنا۔ جب آپ کو ماضی کی کوئی تکلیف دہ بات یاد آ ے	5
					ماضی کی کسی تلخ یاد یا بات سے خود کو بچانا یا بچانے کی کوشش کرنا اور اس کے بارے میں سوچنے یا بات کرنے سے گریز کرنا	6
					ماضی کی کسی تکلیف دہ یاد سے بچنے کے لیے مخصوص سرگرمیوں یا صورتحال سے خود کو بچانا	7
					ماضی کے کسی تکلیف دہ واقعہ کے اہم حصوں کو یاد رکھنے میں مشکل یا پریشانی	8
					اپنے پسندیدہ کاموں یا چیزوں میں دلچسپی کا فقدان	9
					لوگوں سے الگ تھلگ رہنا	10
					اپنوں کے لیے بے حسی یا جزبات کا کم یا ختم ہونا	11
					اپنے مستقبل یا زندگی کے مختصر یا کم ہونے کا ڈر	12
					سونا یا سوئے رہنے میں مسئلہ یا پریشانی	13
					چڑچڑاپن ہونا یا غصہ میں رہنا	14
					توجہ مرکوز نا رہنا یا مشکل ہونا	15
					ہر وقت ہوشیار اور چوکنا رہنا	16
					آسانی سے چونک جانا یا ڈر جانا	17

PTSD CheckList—Civilian Version (PCL-C)

Below is a list of problems and complaints that people sometimes have in response to stressful life experiences. Please read each one carefully and put an "X" in the box to indicate how much you have been bothered by that problem in the last month.

No.	Response	Not at all (1)	A little bit (2)	Moderately (3)	Quite a bit (4)	Extremely (5)
**1.**	Repeated, disturbing memories, thoughts, or images of a stressful experience from the past?					
**2.**	Repeated, disturbing dreams of a stressful experience from the past?					
**3.**	Suddenly acting or feeling as if a stressful experience were happening again (as if you were reliving it)?					
**4.**	Feeling very upset when something reminded you of a stressful experience from the past?					
**5.**	Having physical reactions (e.g. heart pounding, trouble breathing, or sweating) when something reminded you of a stressful experience from the past?					
**6.**	Avoid thinking about or talking about a stressful experience form the past or avoid having feelings related to it?					
**7.**	Avoid activities or situations because they remind you of a stressful experience from the past?					
**8.**	Trouble remembering important parts of a stressful experience from the past?					
**9.**	Loss of interest in things that you used to enjoy?					
**10.**	Feeling distant or cut off from other people?					
**11.**	Feeling emotionally numb or being unable to have loving feelings for those close to you?					
**12.**	Feeling as if your future will somehow be cut short?					
**13.**	Trouble falling or staying asleep?					
**14.**	Feeling irritable or having angry outbursts?					
**15.**	Having difficulty concentrating?					
**16.**	Being "super alert" or watchful on guard?					
**17.**	Feeling jumpy or easily startled?					

PCL-C for DSM IV (11/1/94) Weathers, Litz, Huska, and Keane, National Center for PTSD-Behavioral Science

Division This is a Government document in the public domain.

eReferences

1. Yousufzai M, Harmatz ES, Shah M, Malik MO, Goosens KA. Ghrelin is a persistent biomarker for chronic stress exposure in adolescent rats and humans. *Transl Psychiatry*. 2018;8(1):74.
2. Ford JD, Racusin R, Ellis CG, et al. Child maltreatment, other trauma exposure, and posttraumatic symptomatology among children with oppositional defiant and attention deficit hyperactivity disorders. *Child Maltreat.* 2000;5(3):205-217.
3. Patro BK, Jeyashree K, Gupta PK. Kuppuswamy's socioeconomic status scale 2010-the need for periodic revision. *Indian J Pediatr.* 2012;79(3):395-396.
